# Paediatricians’ perception about oral healthcare of children in Nigeria

**DOI:** 10.1186/s12903-015-0151-2

**Published:** 2015-12-23

**Authors:** Christopher Bismarck Eke, Ezi Abigail Akaji, Oluchi Mildred Ukoha, Vivian Uzoamaka Muoneke, Anthony Nnaemeka Ikefuna, Chika Nwanma Onwuasigwe

**Affiliations:** Department of Paediatrics, College of Medicine, University of Nigeria, Nsukka, Enugu State Nigeria; Department of Community Medicine, College of Medicine, University of Nigeria, Nsukka, Enugu state Nigeria; Department of Paediatrics, Federal Medical Centre, Umuahia, Abia state Nigeria; Department of Preventive Dentistry, College of Medicine, University of Nigeria, Nsukka, Enugu state Nigeria

**Keywords:** Paediatricians’ perception, Oral health care, Children

## Abstract

**Background:**

Majority of the oral diseases in children are preventable. The paediatricians owing to the unique position they occupy in child care are invaluable in achieving standard oral and dental healthcare in children. This study was aimed at assessing the paediatricians’ views on basic oral healthcare in children in Nigeria.

**Methods:**

This was a cross sectional descriptive study. Respondents were paediatricians attending the 2015 Annual General Meeting and Scientific Conference of the Paediatric Association of Nigeria. Relevant information sought from the respondents included: socio- demographic characteristics, duration and location of practice; specific questions on knowledge of basic oral health care in children and recommendations for improvement in knowledge of oral and dental health among paediatricians based on standard clinical guidelines. Data was analyzed using the SPSS version 20.0 and presented in simple frequencies and percentages. Test of associations was done using chi- square while logistic regression analysis was used to determine significantly associated variables (p < 0.05).

**Results:**

A total of 121 paediatricians were recruited. 108 (89.3 %) reported that children should be referred to dental health care when caregivers/older patients report dental problems (*x*^2^ = 9.377; *p* = 0.02). 53.7 % felt that examination of the oral cavity should be routine while 61 (50.4 %) of them reported that health education should be given to caregivers/mothers about nursing caries starting early in life. Majority of the respondents 66 (54.5 %) disapproved gum pad cleaning of young infants. 32 (26.4 %) suggested starting tooth brushing in infants as soon as a tooth appear in a child while 112 (92.6 %) recommended the disapproval of pacifiers in infants/younger children. 93 (76.9 %) were of the opinion that the paediatrician is competent in identifying and handling of basic oral health care in children. However, 108 (89.3 %) recommended that the current postgraduate curriculum in paediatrics should incorporate knowledge of basic oral health care in children.

**Conclusion:**

Majority of the paediatricians were found to have limited knowledge about some basic oral health care in children. It is recommended that the current postgraduate training curriculum in paediatrics in our setting should incorporate knowledge of basic oral healthcare in children.

## Background

Child care physicians by virtue of their duties are responsible for the provision of primary health care to all age groups of children starting from neonatal age through toddlerhood, preschool, school age and adolescence [1]. Further the duties of referring children to other clinical specialties including oral and dental health fall within the whelm of the paediatrician in addition to the provision of preventive oral health education to parents/caregivers as well as older children and adolescents [2, 3]. These the paediatricians contribute towards child dental health because of the unique position they occupy in the society as most children are taken to them to evaluate from an early age in life by their parents/caregivers for medi-care as well as guidance on the ways to ensure that their children’s normal growth and development are met [4].

Majority of the oral diseases in children have been shown to be preventable [5]. And many professional bodies including the American Academy of Pediatric Dentistry (AAPD) have continued to highlight the need for prevention, diagnosis and treatment as key to the restoration and maintenance of oral health of all children [6]. Comprehensive health care therefore can only be realized if dental care is given its right priority in the primary health care.

The importance of prevention, diagnosis and treatment to restore and uphold standard oral health in children therefore cannot be over-emphasized [7]. Paediatricians and other healthcare providers could play an invaluable role in ensuring the maintenance of optimal preventive as well as curative dental health in children [8]. In settings where paediatricians have adequate trainings on oral health care they are invaluable assets to majority of children particularly the poor and minority groups who suffer disproportionately from dental disorders due to limited access to dental care [9].

In Nigeria the introduction of the National Oral Health Policy has opened the door for more interest in oral health particularly in children through the conduct of school oral health screening exercises as well as school based oral health education as part of the school health programme [10, 11].

However, the most important factor affecting preventive oral health performance is the knowledge and function of the medical group, in this instance, the paediatricians concerning oral healthcare in our setting [12]. Several studies have reported limited knowledge of oral health subject matter amongst paediatricians [9, 13–17]. And particularly in Nigeria where the overall dentist/paediatric dental workforce is not the optimal [18], but with a sizeable population of paediatricians spread across the length and breadth of Nigeria [19], it is pertinent to conduct a survey to determine their knowledge of preventive as well as curative dentistry in children. Hence, the aim of the study was to determine paediatricians’ views about oral health care in children in Nigeria.

## Methods

### Study setting

The study was conducted at the 46th Annual General Meeting and Scientific Conference of the Paediatric Association of Nigeria (PANCONF) held at Abakaliki, Ebonyi State, South East Nigeria from 21st to 23rd January, 2015.

### Study design

A cross sectional descriptive study was undertaken. Respondents were consultant paediatricians practicing in Nigeria who attended the 2015 PANCONF and selected consecutively upon obtaining their respective informed verbal consent. A structured questionnaire was designed following the guidelines from American Pediatric Dentistry clinical guidelines, [6] and other previous similar studies [1, 2, 7] and used for the study.

Relevant information sought included bio-data including name, gender, duration of practice as consultant paediatrician, location of practice (Teaching Hospital/Federal Medical Centre/State Specialist Hospital/General Hospital/Others). Others information obtained included specific questions on knowledge of basic oral health care in children and recommendations for improvement in knowledge of oral health among paediatricians.

Ethical approval for the study was obtained from the Health Ethics Research Committee of the University of Nigeria Teaching Hospital, Ituku - Ozalla, Enugu.

### Procedure

All consenting respondents were given a questionnaire respectively at the different scientific sessions during the conference. All completed questionnaires were collected at the close of each scientific session. Two of the researchers ensured that each respondent was sampled only once by adequate record keeping and all questionnaires retrieved at the end of each day’s scientific conference. Trainee members (resident doctors), associate members (including nurses, dieticians and other health workers) and nutrition and pharmaceutical industry staff were excluded from the study. A total of 130 questionnaires were administered out of which 128 were returned with 121 of them having all fields fully completed giving a response rate of 93.1 %.

### Data analysis

Data generated from the study was analyzed using the SPSS version 20.0 (Chicago Il. USA). Simple frequency tables for the characteristics of the respondents were done. Their knowledge of basic oral health care was categorized into “Yes/No” and “approve/disapprove” and routinely/when need arises.

The data were presented in frequencies and percentages. Chi- square statistic was applied to determine the association of the various factors with the level of paediatricians knowledge of basic oral and dental health care including frequency of referral of children to dental healthcare as well as examination of oral cavity during consultation, views on the use of pacifiers in young children, counseling for good oral healthcare, recommendation for gum pad cleaning and when to start tooth brushing in children.

Further statistical analysis was done using the multivariate logistic regression analysis for factors significantly associated with knowledge of basic oral healthcare among the respondents. Here the dependent/outcome variables were: paediatricians’ views on the use of pacifiers (approve or disapprove); recommendation of gum pad cleaning (yes or no); and recommendation on when to start using the toothbrush (correct or incorrect). The independent/Predictor variables were: gender; duration of practice and location of practice.

From the bivariate analysis a p – value of less than 0.16 was chosen arbitrarily and input into the multivariate logistic regression model. The level of statistical significance was set at p < 0.05.

## Results

### Socio- demographic characteristics

Majority of the study participants were females 66 (54.5 %) and newly qualified paediatric consultants, 60 (49.6 %) who had between one to five years of practice experience respectively. Eighty one percent were working in Government hospitals (including Teaching Hospitals/Federal Medical Centres/State Specialist Hospitals. The socio-demographic characteristics of the respondents are as shown in Table 1.

**Table 1:** Demographic characteristics of respondents

Characteristic	(n = 121)	(%)
Sex:		
Male	55	45.5
Female	66	54.5
Duration of practice (in years):		
1 – 5	60	49.6
>5 – 10	17	14.0
>10	44	36.4
Location of Practice:		
Teaching Hospital/FMC/State Specialist Hospital	98	81.0
General Hospital	14	11.6
Private Specialist Hospital	5	4.1
Others	4	3.3

Key: *FMC* Federal Medical Centre, Others- Faith – Based Hospital

### Knowledge about oral and dental health care among paediatricians

One hundred and eight (89.3 %) reported that children should be referred to dental health care when caregivers/older patients report dental problems while 13 (10.7 %) felt that it should be routine. The difference was statistically significant (*x*^2^ = 9.377; *p* = 0.02). 53.7 % felt that examination of the oral cavity should be routine while 61 (50.4 %) of them reported that health education should be given to caregivers/mothers about nursing caries starting early in life.

Majority of the respondents 66 (54.5 %) disapproved gum pad cleaning of young infants.

A few of the respondents 32 (26.4 %) in this study suggested starting tooth brushing in infants as soon as a tooth appear in a child and nearly one-fifth (19.8 %) were ambivalent of the correct time to initiate tooth brushing in a child. However, 53 (43.8 %) recommended starting of tooth brushing after a few teeth appeared in infants. There was a statistically significant difference between knowledge of when to start tooth brushing after a few teeth appear in infants and location of practice (*x*^2^ = 18.551; *p* = 0.029). Nearly all the respondents, 119 (98.3 %) had access to dentists for referral in their places of practice.

In all, majority of the studied paediatricians, 93 (76.9 %) were of the opinion that the paediatrician is competent in identifying and handling of basic oral health care in children compared to 23 (28.1 %) that felt differently.

Majority of the respondents, 112 (92.6 %) recommended the disapproval of pacifiers in infants/younger children. And the comparison of the paediatricians’ views about use of pacifiers in infants according to the duration of practice (in years) was statistically significant (*x*^2^ = 6.363; *P* = 0.042) as shown in Table 2 as well as location of practice (*x*^2^ = 13.605; *p* = 0.003); see Table 3. Similarly, 66.1 % of respondents considered thumb sucking habit harmful.

**Table 2:** Relationship of paediatricians’s knowledge of early childhood oral health activities to duration of practice as a specialist

	Duration of practice as specialist						
Knowledge question item:	Responses	1-5 yrs n = 60(%)	6-10 n = 17(%)	≥10 n = 44(%)	Total n = 121(%)	×^2^	P – value
Frequency of referral of patients (children) to dental health care:							
	Routinely	4(30.8)	2(15.4)	7(53.8)	13(100.0)	2.194	0.334
	When caregivers report dental problems	55(50.9)	16(14.8)	37(34.3)	108(100.0		
Frequency of examination of the oral cavity during consultations:	Routinely	23(41.1)	9(16.1)	24(42.9)	58(100.0)	2.573	0.276
	Only when indicated by history	36(55.4)	9(13.8)	20(30.8)	65(100.0)		
Health education to mothers on prevention of caries	No	28(46.7)	8(13.3)	24(40.0)	60(100.0)	0.730	0.694
	Yes	31(50.8)	10(16.4)	20(32.8)	61(100.0)		
View on the use of pacifiers in infants	Approve	8(88.9)	0(0.0)	1(11.1)	9(100.0)	6.363	0.042
	Disapprove	51(45.5)	18(16.1)	43(38.4)	112(100.0)		
Routine counseling for good oral health maintenance of caregivers/older children	No	8(42.1)	3(15.8)	8(42.1)	19(100)	0.422	0.810
	Yes	51(50.0)	15(14.7)	36(35.3)	102(100.0)		
Recommendation of gum pad cleaning in young children	No	20(45.5)	12(18.2)	24(36.4)	66(100.0)	1.392	0.499
	Yes	29(52.7)	6(10.9)	20(36.4)	55(100.0)		
Recommendation on when to start tooth brushing in a child							
	As soon as a tooth appears	19(57.6)	2(6.1)	12(36.4)	33(100.0)	3.667	0.722
	After a few teeth appear	23(43.4)	10(18.9)	20(37.7)	53(100.0)		
	When a child can hold a tooth brush	6(54.5)	2(18.2)	3(27.3)	11(100.0)		
	Ambivalent (Not Sure)	11(45.6)	4(16.7)	9(37.5)	24(100.0)

**Table 3:** Relationship of paediatricians’ knowledge of early childhood oral health activities to location of practice as specialist

Knowledge question item:	Responses	Teaching Hosp/FMC/State specialist hospital	General hospital	Private specialist hospital	Others	Total	×^2^	P-value
	Routinely	8(61.5)	0(0.0)	3(23.1)	2(15.4)	13(100.0)	9.377	0.025
Frequency of referral of your patients (children) to dental healthcare	When caregivers report dental problems	90(83.3)	5(4.6)	11(10.2)	2(1.9)	108(100.0)		
How often do you examine the oral cavity	Routinely	46(82.1)	2(3.6)	4(7.1)	4(7.1)	56(100.0)	6.505	0.089
	only when implicated by history	52(80.0)	3(4.6)	19(15.4)	0(0.0)	65(100.0)		
Do you give mothers health education on prevention of caries	No	49(81.7)	2(3.3)	9(15.0)	0(0.0)	60(100.0)	5.335	0.149
	Yes	49(80.3)	3(4.9)	5(8.2)	4(6.6)	61(100.0)		
What is your view on use of pacifiers in infants	Approve	4(44.4)	2(22.2)	3(33.3)	0(0.0)	9(100.0)	12.605	0.003
	Disapprove	94(83.9)	3(2.7)	11(9.8)	4(3.6)	112(100.0)		
Do you recommend gum pad cleaning in young infants	No	58(87.9)	3(4.5)	4(6.1)	1(1.5)	66(100.0)	6.128	0.106
	Yes	40(72.7)	2(3.6)	10(18.2)	3(5.5)	55(100.0)		
Recommendation on when to start tooth brushing in a child	As soon as a tooth appears	25(75.8)	0(0.0)	4(12.1)	4(12.1)	33(100.0)		
	After a few teeth appear	46(86.8)	2(3.8)	5(9.4)	0.(0.0)	53(100.0)	18.551	0.021
	When a child can hold a tooth brush	8(72.7)	2(18.2)	1(9.1)	0(0.0)	11(100.0)		0.029
	Not Sure	19(79.2)	1(4.2)	4(16.7)	0(0.0)	24(100.0)		
Do you routinely give counseling for good oral health maintenance to caregivers/older children	No	18(94.7)	0(0.0)	1(5.3)	0(0.0)	19(100.0)	2.977	0.395
	Yes	80(78.4)	5(4.9)	13(12.7)	4(3.9)	102(100.0)		

However, 108 (89.3 %) recommended that the current postgraduate curriculum in paediatrics should incorporate knowledge of basic oral health care in children.

Bivariate analysis of the paediatricians’ views on the use of pacifiers against gender, duration of practice and location of practice yielded p-values of 0.508, 0.153 and 0.131 respectively. Similarly bivariate analysis of the paediatricians’ recommendation of gum pad cleaning against gender, duration of practice and location of practice yielded p-values of 0.463, 1.000 and 0.013 respectively. Also, the bivariate analysis of the paediatricians’ recommendation of when to start tooth brushing against gender, duration of practice and location of practice yielded p-values of 0.442, 0.782 and 0.138 respectively.

It was then decided to input the variables with the p-values of less than 0.16 into the multivariate logistic regression model which showed that paediatricians’ with more than 10 years duration of service were about six times more likely to approve the use of pacifiers while those that were practicing in the private health facilities were less likely to approve the use of pacifiers. These associations were however not statistically significant. Paediatricians’ that practiced in the private health facilities were significantly less likely to recommend gum pad cleaning in children. Also, the paediatricians’ that practiced in the private health facilities were about two times more likely to recommend the correct time to start using toothbrush. However, the association was not statistically significant. See Table 4 (A to C).

**Table 4:** Multivariate Analysis of the Paediatricians’ Knowledge of Oral Healthcare in children

Predictor variable	p-value	Odds ratio	95 % CI for odds ratio	
			Low	Upper
A. Paediatricians’ Views on Use of Pacifiers				
Duration of practice (Reference variable: ≤ 10 years )	0.096	6.283	0.721	54.766
Location of practice (Reference variable: Public health facility)	0.067	0.231	0.048	1.107
B. Paediatricians’ Recommendation on Gum Pad Cleaning				
Location of practice (Reference variable: Public health facility)	0.018	0.265	0.088	0.799
C. Paediatricians’ Recommendation on when to Start Using Toothbrush				
Location of practice (Reference variable: Public health facility)	0.145	2.269	0.754	6.828

## Discussion

The paediatricians by virtue of their training and services they render are the first set of medical personnel responsible for evaluating every neonate and subsequently continue to take charge of their healthcare till the child attains the official age of maturity [1, 4, 7]. The paediatrician therefore occupies a central and important position towards the realization of comprehensive healthcare for all children including oral health care. However, from the current study majority of the respondents (paediatricians) were found to have limited knowledge about most standard oral health care in children. Several studies have reported poor knowledge of oral health subject matter amongst paediatricians [9, 13–17]. However studies from Turkey [20], as well as in Canada [21], and some other workers had reported contrary views [9, 22].

The training of a qualified paediatrician in Nigeria currently requires 3 stages of examinations including primaries, parts I and II fellowship examinations lasting between 4 to 6 years. During the periods of parts I and II postings every trainee is exposed to various core and elective postings in addition to general paediatrics. However, postings in dentistry/paediatric dentistry in the undergraduate as well as post graduate training in paediatrics in addition to continuing medical education in such important subject matter like paediatric oral health are currently lacking in our setting and this could be partly responsible for the poor knowledge of current preventive pediatric oral healthcare among paediatricians.

Concerning the practice of paediatric dentistry particularly in the United States, the American Academy of Paediatric Dentistry [6, 7], had recommended early referral of every child to see a paediatric dentist from six months of age. From the present study, in terms of early referral for dental consultations, most of the respondents favored referral of a child for a dental consultation following caregiver/older child complaint of dental problems. The need for early referral in order to maintain standard oral healthcare in children cannot be over- emphasized as it helps to achieve and sustain standard oral healthcare in our children.

In some settings where dentists and particularly paediatric dentists are limited or in impoverished communities, a paediatrician with adequate dental exposure during medical school as well as residency training could be of invaluable asset [9]. Though the paediatric workforce in Nigeria is still not the desired [19], the current geographical spread is still offering a reasonable level of care to our children nationwide and the bulk of their basic oral health care are most likely anchored on the paediatricians.

Majority of the paediatricians disapproved gum pad cleaning in young infants. However most researchers have demonstrated that breastfeeding could be associated with caries formation particularly when the feeding pattern is associated with libitum feeding known to promote tooth decay and dental carries [23]. However, it has been shown that regular cleaning of the gum pad with moist gauzed pad/clean washcloth could help in reducing the accumulations of bacteria and other micro-organisms in the month which could convert milk sugar (lactose) or other fermentable carbohydrates to acids and predispose the child to formation of dental caries [7]. Exclusive breastfeeding is known to be the bedrock of infant nutrition. However, the exclusive breastfeeding rate in Nigeria is as low as 17 % [24], which implies that most of the children are either practicing partial breastfeeding and another few on exclusive formula feeding which promotes the likelihood of formation of caries. Though majority of the study participants may have responded contrary to the best practice guidelines mothers should be counseled on wiping of their babies gum with clean, moist gauze pad or washcloth every day to limit the development of caries in them [7, 25].

In furtherance to the assurance of adequate oral health in children, caregivers ought to be educated and meant to also understand when they should commence tooth brushing in children. A few of the respondents 32 (26.4 %) in the current study suggested starting tooth brushing in infants as soon as a tooth appear in a child and nearly one-fifth (19.8 %) were ambivalent of the correct time to initiate tooth brushing in a child. The importance of early initiation of tooth brushing in children as soon as a tooth appears cannot be over-emphasized as it reduces the incidence of carries as well as improves the overall oral health. Timely initiation of tooth brushing in a child equally ensures sustainability of good tooth brushing later in childhood and adulthood respectively [26].

Majority of the respondents disapproved the use of pacifiers in infants and young children. Some oral habits including pacifier usage are common in young children and have been reported to help in preventing mortality due to sudden infant death syndrome [27]. Though it has some desirable effects, pacifier usage in children if used more frequently and prolonged could equally predispose such children to development of otitis media [28], delayed speech development [29], in addition to some dental disorders like dento- alveolar deformities [29, 30]. Hence, it is recommended that further paediatric neurological as well as dental evaluation be sought if non-nutritive sucking habits persist beyond three years of age [31]. Therefore the benefit of the use of pacifier in young children should be weighed against its possible health risks and erred on the side of caution.

Most of the respondents (76.9 %) felt that the paediatricians by general and our local content training are competent in terms of the provision of preventive oral health as well as identifying cases requiring dental expertise which are referred accordingly in our setting.

However, nearly four-fifth of them recommended that the current postgraduate training curriculum in paediatrics should incorporate knowledge of basic oral healthcare in Nigeria. The American Academy of Paediatrics (AAP) has put up various educational training programmes including online module training curricular for continuing medical education for physicians aimed at improving their quality of care of the children accessing healthcare in their homeland [8]. Such learning programmes could be adopted and reviewed by the Paediatric Association of Nigeria (PAN) and made available to its members with the view of increasing capacity in the knowledge of oral healthcare in children among practicing paediatricians in Nigeria.

## Conclusion

From the current study majority of the paediatricians were found to have limited knowledge about some basic oral health care in children. It is recommended that the current postgraduate training in paediatrics in our setting should incorporate knowledge of basic oral healthcare in children.

## Abbreviations

AAP: American Academy of Paediatrics
AAPD: American Academy of Pediatric Dentistry
FMC: Federal Medical Centre
NGO: Non- Governmental Organization Hospital.
PANCONF: Paediatric Association of Nigeria Annual General Meeting and Scientific Conference
PAN: Paediatric Association of Nigeria
WHO: World Health Organization
